# Emission of Per- and Polyfluoroalkyl Substances from a Waste-to-Energy Plant—Occurrence in Ashes, Treated Process Water, and First Observation in Flue Gas

**DOI:** 10.1021/acs.est.2c08960

**Published:** 2023-06-15

**Authors:** Sofie Björklund, Eva Weidemann, Stina Jansson

**Affiliations:** † Department of Chemistry, Umeå University, SE-901 87 Umeå, Sweden; ‡ Industrial Doctoral School, Umeå University, SE-901 87 Umeå, Sweden

**Keywords:** PFASs, waste incineration, bottom ash, fly ash, municipal solid waste

## Abstract

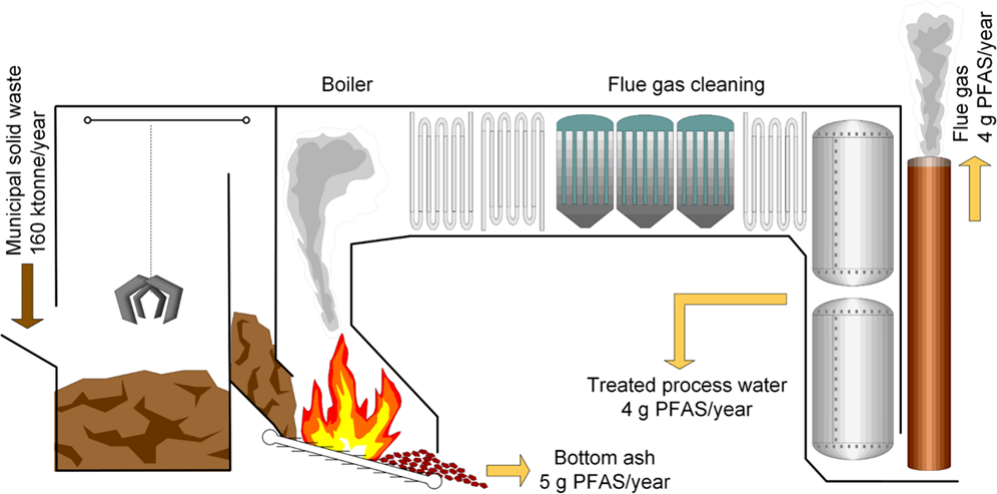

Per- and polyfluoroalkyl substances (PFASs) are a large group of compounds commonly used as industrial chemicals and constituents of consumer products, e.g., as surfactants and surface protectors. When products containing PFASs reach their end of life, some end up in waste streams sent to waste-to-energy (WtE) plants. However, the fate of PFASs in WtE processes is largely unknown, as is their potential to enter the environment via ash, gypsum, treated process water, and flue gas. This study forms part of a comprehensive investigation of the occurrence and distribution of PFASs in WtE residues. Sampling was performed during incineration of two different waste mixes: normal municipal solid waste incineration (MSWI) and incineration of a waste mix with 5–8 wt % sewage sludge added to the MSWI (referred to as Sludge:MSWI). PFASs were identified in all examined residues, with short-chain (C4–C7) perfluorocarboxylic acids being the most abundant. Total levels of extractable PFASs were higher during Sludge:MSWI than during MSWI, with the total annual release estimated to be 47 and 13 g, respectively. Furthermore, PFASs were detected in flue gas for the first time (4.0–5.6 ng m^–3^). Our results demonstrate that some PFASs are not fully degraded by the high temperatures during WtE conversion and can be emitted from the plant via ash, gypsum, treated process water, and flue gas.

## Abstract 2

Minimal research exists on the fate of per- and polyfluoroalkyl substances (PFASs) in waste-to-energy processes. This study, the first of its kind, depicts a snapshot of the presence of PFASs in waste-to-energy residues.

## Introduction

Per- and polyfluoroalkyl substances (PFASs) are a diverse group of compounds characterized by highly fluorinated alkyl chains and strong C–F bonds, which endow them with surfactant properties and resistance to physical and chemical degradation.^1^ These characteristics make PFASs attractive for a large number of applications, e.g., as industrial chemicals or constituents of consumer products, including surface protection of textile and leather products, food contact materials, and nonstick products.^2^ Moreover, they commonly occur in construction materials, such as composite wood building materials, floor coverings, and insulation materials.^2,3^

Given their widespread usage, some products containing PFASs will at their end of life inevitably end up in waste streams. The most common method of waste disposal on a global scale is landfilling,^4^ and many studies have shown the presence of PFASs in leachate from landfills.^5−9^ However, other forms of waste management, such as waste-to-energy (WtE) facilities, may be secondary release routes of PFASs.^9−12^ Quantitative estimations of PFASs released from WtE plants are scarce but have shown that PFASs can leach from waste prior to incineration.^10−12^ Moreover, some studies have reported the presence of PFASs in WtE ashes.^9,11,13^

Therefore, the role of WtE residues as a sink for hazardous substances needs to be assessed to minimize the risk of them being a secondary source of PFASs into the environment. What happens to PFASs during incineration in a full-scale WtE plant is largely unknown. One study by Liu et al. detected PFASs in fly ash and bottom ash from municipal solid waste (MSW) incineration in China.^11^ Another study compared PFASs levels in leachate from three landfill sites in the U.S. receiving ash from MSWI plants operating at different temperatures.^9^ An overall trend of decreasing total amounts of PFASs in leachate with increasing incineration temperature was observed,^9^ although the landfills contained different ratios of ash and MSW (65, 98, and 100% ash, respectively), making direct comparison difficult.

Owing to the limited number of studies available, more comprehensive investigations into the fate of PFASs in full-scale WtE plants are needed.^14,15^ One notable knowledge gap is that existing studies have exclusively focused on ashes,^9,11^ while neglecting liquid and gaseous residues. There is also a substantial divergence between existing lab-scale studies regarding the degradation efficiency under typical waste incineration conditions. These divergencies may be due to a range of factors, including incomplete lists of potential byproducts, varying combustion conditions, and the general complexity of incineration chemistry.^16,17^ Importantly, the potential role of WtE flue gases as a release vector of PFASs remains to be investigated. Ideally, a study investigating the fate of PFASs in WtE should include a complete mass balance, covering all parts of the process, from PFASs entering via the waste, to the residual streams leaving the facility. This would enable an estimation of total release of PFASs from WtE facilities. However, in addition to a systematic sampling design and validated analytical protocols, such a study would require an extensive sampling and analysis effort of the waste fuel. Given that full-scale WtE production lines incinerate hundreds of tons of waste per day, logistic challenges alone (i.e., storing, crushing, quartering) make it extremely difficult to conduct feedstock analysis reliably. Representativity of the samples is difficult to achieve and not possible to conduct as part of this study. The major challenges related to obtaining the mass balance are the analytical sample size (i.e., grams) in comparison to the amount of waste incinerated on an hourly basis (i.e., tons), and the need to collect, store, sample, quarter, and prepare representative samples from the waste fuel, considering that the majority of the fuel is delivered directly to the waste bunker.

This study is part of an extensive sampling campaign of a full-scale WtE facility to examine the importance of WtE residues as secondary release routes of PFASs. The specific aim for this part of the investigation was to examine the residual fractions leaving the facility, i.e., bottom ash, flue gas cleaning residues, gypsum, treated process water, and flue gas, to determine the distribution and types of PFASs in the residues. Moreover, by establishing the PFAS concentrations of all fractions leaving the WtE facility, an estimate of the total annual release of PFASs could be conducted.

## Materials and Methods

Standards of 18 native PFASs, including C4–C14 perfluorocarboxylic acids (PFCAs), C4–C12 perfluorosulfonic acids (PFSAs), fluorotelomer sulfonic acids (FTSAs), and polyfluoroalkyl phosphoric acid diesters (diPAPs) as well as 9 isotope-labeled PFASs (see full list in Table S2) were obtained from Wellington Laboratories (Guelph, ON, Canada). Methanol was obtained from Fisher Scientific (Leicestershire, U.K.), ammonium acetate from Fluka (Buchs, Switzerland), acetic acid from Merck (Darmstadt, Germany), ammonium hydroxide and hydrochloric acid from J.T. Baker (Phillipsburg, NJ), and sodium hydroxide from VWR International (Radnor, PA). Ultrapure water was prepared using a Milli-Q Advantage system (Millipore, Billerica). Weak anion exchange solid-phase extraction (WAX-SPE) cartridges (Oasis WAX, 6 cc, 150 mg sorbent, 30 mm particle size) were purchased from Waters Corp. (Milford, MA). Activated carbon disks (47 mm × 2 mm) were supplied by Futamura Chemical Co., Ltd. (Nagoya, Aichi, Japan) and GF/A filters (47 mm diameter, pore size 1.6 μm) from VWR International (Radnor, PA).

### Site Description

Samples were collected at a full-scale WtE plant in northern Sweden. The plant incinerated on average 20 tonnes of waste per hour in a moving grate boiler, producing up to 50 MW of district heating and 15 MW of electricity. The plant is a state-of-the-art facility with absolute compliance with legislative demands on incineration temperature, residence time, and emission limits. The waste fuel was mainly a mix of residual waste from households (i.e., waste after recyclables and food waste have been sorted out by households) (60%) and industrial waste (e.g., discarded furniture, construction, and demolition waste) (40%). The majority of the waste fuel was placed in the waste bunker directly when arriving at the facility, while a small portion was pretreated by, e.g., crushing overly large pieces. The waste fuel was incinerated at a minimum temperature of 850 °C for 2 s, but grate temperatures could reach 1100 °C under normal operation. More detailed descriptions of the plant and the incineration parameters are given in Section S1.

### Study Design

Sampling was performed during two campaigns consisting of 3 days each: one sampling campaign where the waste fuel mix was incinerated as received (referred to as municipal solid waste incineration, MSWI) and one where wastewater treatment sludge (15–20 wet wt %) was mixed with the waste fuel at ca. 5–8% on wet basis (referred to as Sludge:MSWI). Because the exact characteristics of the incinerated waste at a given timepoint were unknown, the addition of sewage sludge provided a case in which a material known to contain PFASs was included in the fuel mix.^18−20^

**Figure 1:**
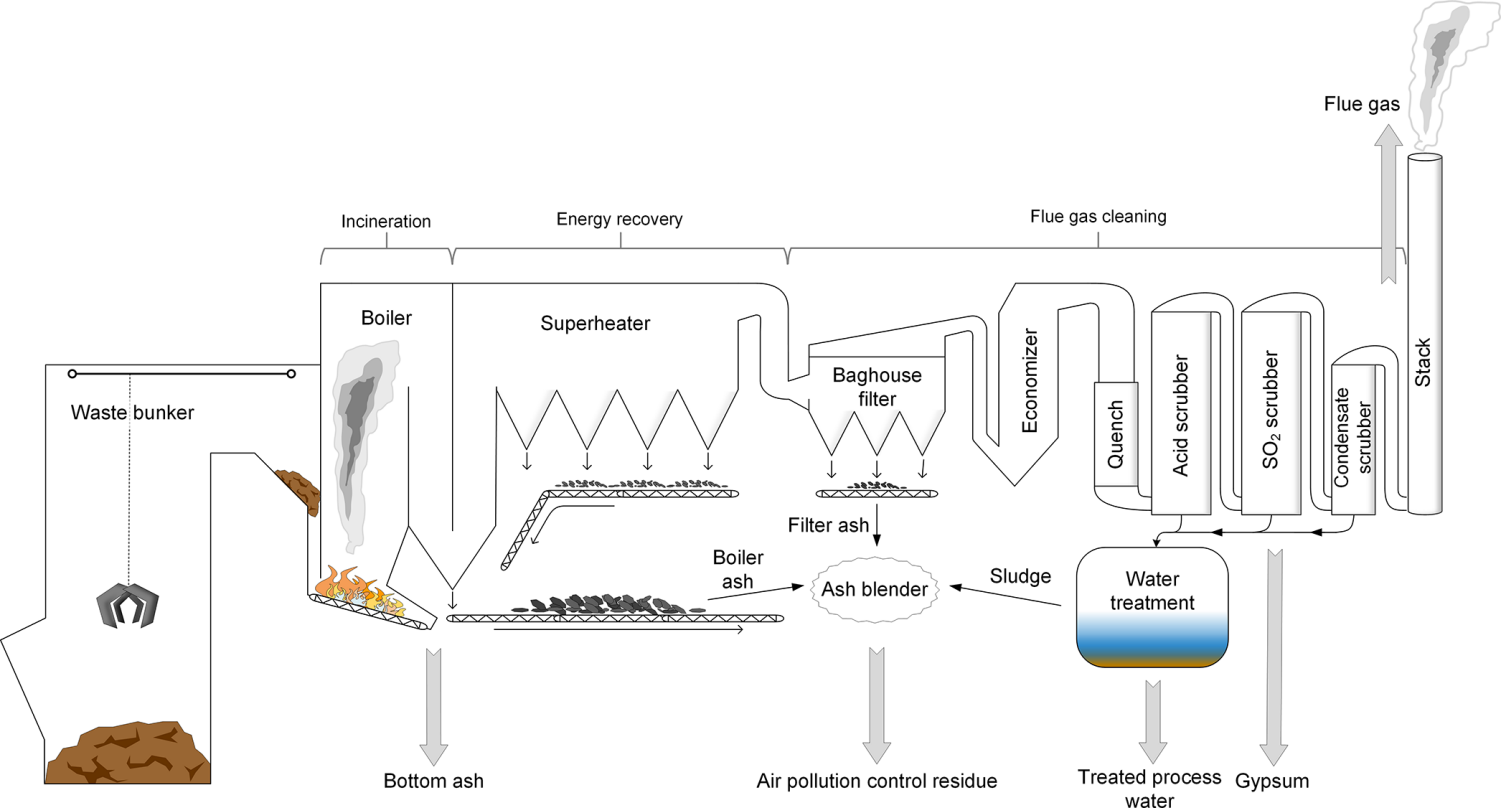
Overview of the waste incineration plant, with sampling points indicated by gray arrows.

Treated process water (i.e., a combination of condensate, acid scrubber water, and a small fraction of process chemicals, Figure 1 and Section S1) was collected on three occasions per sampling day and pooled. Bottom ash, air pollution control residue (APCR, i.e., a mixture of fly ash and sludge from the WtE water treatment, Figure 1 and Section S1), and gypsum were collected at the end of each sampling day. Flue gases were sampled in the stack using the EN 1948:1 sampling train, modified with additional features from air sampling methodologies. Briefly, the sampling train consisted of a cooled probe, followed by two impinger bottles containing 250 mL Milli-Q and 200 mL 0.1 M sodium hydroxide, respectively, and an activated carbon disk mounted between two polyurethane foam plugs. Prior to the sampling campaigns, in-house testing of the flue gas sampling device was conducted using spiked solutions to verify efficient retention of PFASs substances. Flue gas sampling was performed for 6 h at a flow rate of 16 L min^–1^. Additional details are provided in Section S2.

### Sample Preparation

Extraction of solid samples was conducted based on a protocol developed by the Swedish Environmental Research Institute IVL,^21^ with minor modifications. Bottom ash was ball-milled for 5 min to crush large pieces prior to extraction. For bottom ash, APCR, and gypsum, a subsample of 1–2 g was weighed into a 15 mL polypropylene tube and spiked with 3 ng of isotope-labeled standard. After adding 5 mL of methanol, the sample was vortexed for 10 s and ultrasonicated for 15 min. The sample was then centrifuged at 1200*g* for 10 min, and the supernatant was transferred to a new tube. The extraction was repeated once, after which the extracts were combined, and the volume reduced under a nitrogen flow to 1 mL. The extract was diluted to 10 mL with ultrapure Milli-Q water, and the pH was adjusted to 4 using acetic acid.

Subsamples of treated process water (∼250 mL) were spiked with 3 ng of isotope-labeled standard, and the pH was adjusted to 4 using acetic acid. The treated process water was then extracted by WAX-SPE using the protocol described below.

For flue gas samples, the Milli-Q phase, sodium hydroxide phase, and filters were extracted and analyzed separately. All compartments were spiked with 3 ng of isotope-labeled standard each. The filters were extracted according to the same procedure as the solid samples but using 50 mL methanol. The Milli-Q and sodium hydroxide phases were adjusted to pH 4 using acetic acid and hydrochloric acid, respectively, and extracted by WAX-SPE, as described below.

All samples were extracted by WAX-SPE according to the ISO 25101 methodology, with minor modifications described elsewhere.^10^ The cartridges were preconditioned with 4 mL of 0.1% ammonium hydroxide in methanol, followed by 4 mL of methanol and 4 mL of Milli-Q water. Samples were loaded at a rate of ∼1 drop per second and rinsed with 4 mL of 25 mM ammonium acetate buffer and 4 mL of Milli-Q and then dried for 30 min under vacuum. Samples were eluted in two fractions of 4 mL of methanol, followed by 4 mL of 0.1% ammonium hydroxide in methanol. The extracts were evaporated to 150 μL under a gentle stream of nitrogen and transferred with methanol to a LC-vial with a final volume of 0.5 mL. A recovery standard was added, and 80 μL of the extract was combined with 120 μL of 2 mM ammonium acetate in a vial for analysis.

### LC-MS Analysis

The analysis was performed on a 6560 Ion Mobility Q-ToF LC-MS (Agilent Technologies) with electrospray ionization operating in the negative mode. Separation was performed on a C18 column (3 μm, 110 Å, 150 × 2.0 mm^2^, Phenomenex, Torrance, CA) using a 0.5 mL flow rate and water–methanol gradient (both containing 2 mM ammonium acetate), starting at 30% methanol and holding for 2 min, then increasing to 100% methanol over 12 min, before finally holding at 100% methanol for 3 min (additional details in Tables S2, S11, and S12).

### Quality Control

Quantification was performed using internal calibration with corresponding isotopically labeled standards. For compounds lacking isotope-labeled standards, the internal standard closest in retention time was used. The limit of detection (LOD) was calculated as the average procedural blank level plus 3 times the standard deviation. If no PFASs were detected, LOD was calculated as 3 times the instrument noise level (Table S4). The limit of quantification (LOQ) was calculated as 10 times the instrument noise level. Mass error for all quantified compounds was less than 7 ppm.

Water samples were collected in high-density polyethylene (HDPE) flasks and stored at −18 °C upon arrival at the lab. Solid samples were collected in stainless steel containers, and subsamples were transferred to polypropylene (PP) tubes after quartering.

For flue gas sampling, an isotope-labeled standard was added to the Milli-Q phase prior to sampling. Polyurethane foam filters and activated carbon disks were washed in methanol for at least 12 h before sampling. Sample flasks were precleaned by baking at 550 °C for 5 h and thoroughly rinsed with methanol before sampling. To facilitate comparison, the volume of flue gas sampled was normalized to that of dry gas, 0 °C, and a pressure of 1 atm (additional details in Section S2).

Field blanks were collected for each sampling campaign. Generally, no PFASs were detected, with some exceptions (Table S6). Two procedural blanks consisting of Milli-Q water were included in every batch of samples, giving a total of 18 procedural blanks (Table S7). When sample quantity allowed, triplicate or duplicate samples were included. The average relative standard deviation of all replicate samples was 12 ± 11% (Table S5).

## Results and Discussion

### PFASs in WtE Residues

**Figure 2:**
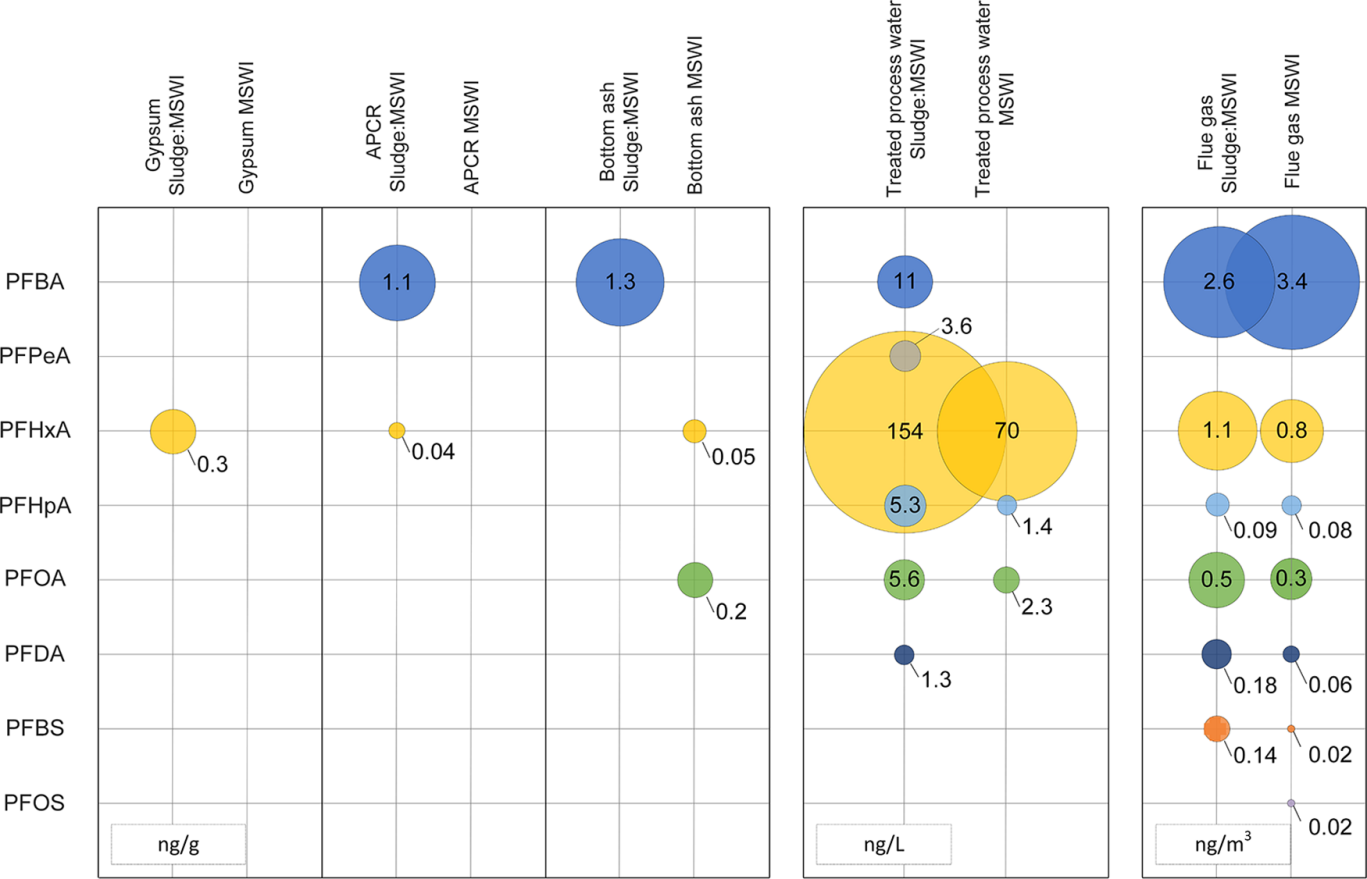
Content of PFASs in waste incineration residues. Circle radii denote the average concentration in the sample matrix. APCR: air pollution control residue.

PFASs were found in all residual streams. Overall, eight individual PFASs were detected and short-chain PFCAs (C4–C7), mainly PFBA and PFHxA, were dominant. In addition, PFBS and PFOS were detected. Total levels of detected extractable PFASs were generally higher in incineration residues generated from Sludge:MSWI than from MSWI (Figure 2 and Table S3).

### MSWI

The total concentration of extractable PFASs determined in treated process water was 62–97 ng L^–1^ (Table S3). Only PFCAs were detected, of which PFHxA represented 93–97% on a ng L^–1^ basis. Other PFCAs detected were PFHpA (n.d. to 2.5 ng L^–1^) and PFOA (1.7–3.2 ng L^–1^).

To the best of our knowledge, no other study has investigated the presence of PFASs in treated process water from waste incineration plants. However, some studies have reported finding PFASs in leachate from municipal solid waste storage sites prior to incineration.^10−12^ In leachate from a temporary waste stockpile at the same WtE plant studied herein, the total concentration of extractable PFASs was 170–260 ng L^–1^.^10^ The same study found that short-chain PFCAs made up 67% of the total concentration, whereas the remaining 33% comprised equal amounts of PFSAs and long-chain PFCAs. In a study by Wang et al., concentrations of PFASs (C4-C11) in leachate from two MSW incineration plants and two waste transfer stations in Tianjin, China, ranged from 4700 to 28 100 ng L^–1^.^12^ Moreover, the relative abundance of different groups of PFASs varied between the different locations. For example, in leachate at the waste transfer stations, PFCAs represented ∼20% of the total concentration, whereas in leachate from the MSW incineration plants, they made up 50–75%. In another study of leachate from MSWI plants in Shenzhen, China, total extractable PFAS levels were even higher at 21 000–682 000 ng L^–1^.^11^ There, PFSAs made up a larger fraction (50–80%) of total extractable PFASs compared to other studies. In a report by the Swedish Research Institute IVL, condensate from 24 Swedish WtE plants was found to contain mainly PFCAs at concentrations of 0.28–180 ng L^–1^.^21^ Similarly to the present study, short-chain PFCAs were the most abundant group of PFASs detected.

In our study, no PFASs were detected in gypsum or APCR during MSWI. In bottom ash, PFHxA was detected in only one sample, at a concentration of 0.16 ng g^–1^, and another sample contained PFOA, at a concentration of 0.54 ng g^–1^. Solo-Gabriele et al. recently investigated the occurrence of PFASs in landfill leachates from ash monofills containing bottom and fly ash generated from MSWI at 760–980 °C.^9^ In agreement with the present study, short-chain PFASs were found to be most prevalent regardless of the incineration temperature, representing ∼60% of the total extractable PFASs concentration. However, a significant fraction of the detected PFASs (25–30%) comprised PFSAs, which were not detected in bottom ash or APCR in the present study.

In the flue gas sampled within our study, PFBA was the most abundant PFAS (2.8–3.8 ng m^–3^), followed by PFHxA (0.22–1.9 ng m^–3^). Moreover, PFOA was detected at levels of 0.16–0.40 ng m^–3^. PFHpA and PFOS were detected in one sample at 0.23 and 0.07 ng m^–3^, respectively, whereas PFDA was detected in two samples (0.05 and 0.13 ng m^–3^). In the same two samples, PFBS was detected at relatively low concentrations (0.03 ng m^–3^). To the best of our knowledge, this is the first time the EN 1948-1 sampling protocol has been modified for PFAS sampling. Before the sampling method is fully adapted for PFAS determination in flue gases, further validation is needed.

Although no previous study has reported on the occurrence of PFASs in flue gas from a full-scale WtE plant, air samples collected at a MSWI plant in China have been examined.^12^ Like in the present study, the majority of PFASs detected were PFCAs (88–94%). However, long-chain PFASs were detected in relatively higher abundance, representing 36–60% of the total concentration vs 5–18% in the present study. This could be due to the influence of non-incinerated MSW stored at the plant. However, differences should be interpreted with caution since the abovementioned study used a passive sampling method.

### Sludge:MSWI

During the sampling campaign in which sludge was added to the waste fuel mix, total extractable PFAS concentrations in the treated process water ranged from 160 to 220 ng L^–1^. Similarly to MSWI, PFHxA was most abundant (132–190 ng L^–1^). PFOA was present at 4.3–6.6 ng L^–1^ and PFHpA at 4.0–7.6 ng L^–1^. PFBA (8.8–13 ng L^–1^), PFPeA (3.0–4.3 ng L^–1^), and PFDA (0.94–1.6 ng L^–1^) were detected in all samples. Compared to MSWI, the average total concentration of extractable PFASs in treated process water was more than twice as high during Sludge:MSWI than during MSWI (74 ± 16 ng L^–1^ vs 180 ± 28 ng L^–1^).

Whereas no PFASs were detected in gypsum during the MSWI samplings, PFHxA was detected at concentrations of 0.17–0.31 ng g^–1^ during Sludge:MSWI. The gypsum produced by the incineration process originated from the SO_2_ scrubber (Figure 1), where the flue gases are showered with a Ca(OH)_2_ slurry, which reacts with SO_2_ in the flue gases to form gypsum (CaSO_4_). Therefore, PFASs found in gypsum can be traced back to either the flue gas passing through the SO_2_ scrubber or the Ca(OH)_2_. In this case, as no PFASs were detected in the MSWI gypsum, it is plausible that the PFASs in the Sludge:MSWI case originated from the flue gases.

In APCR, total concentrations of extractable PFASs ranged from 0.99 to 1.3 ng g^–1^. PFBA was detected in all samples, whereas PFHxA was detected in only one sample. In comparison, no PFASs were detected during MSWI. In a previous study of fly ash from three MSW incineration plants in China, total levels were substantially higher, ranging from 1.5 to 77 ng g^–1^.^11^ In the same study, PFOS was the predominant PFAS at two of the three plants, whereas PFBA was most prevalent at the third plant. The latter plant also had the lowest mean concentration of PFASs in the study (4.0 ng g^–1^ compared to 13 and 33 ng g^–1^).^11^

In bottom ash, PFBA was the only detected PFAS, ranging from 0.81 to 1.5 ng g^–1^. In contrast, PFHxA and PFOA were detected during MSWI (0.16 and 0.54 ng g^–1^, respectively). As in APCR, concentrations detected in bottom ash in the present study were considerably lower than those found by Liu et al., where average levels ranged from 10 to 17 ng g^–1^.^11^ In the same study by Liu et al., short-chain PFCAs were found to be dominant at two out of three investigated plants (45 and 86% respectively), whereas PFSAs were most prevalent at the third plant (57%).

In the sampled flue gas, PFBA was found to be most abundant, followed by PFHxA and PFOA (2.5–27, 0.39–2.0, and 0.23–0.79 ng m^–3^, respectively). Moreover, PFDA and PFBS were detected in all samples (0.10–0.23 and 0.07–0.24 ng m^–3^, respectively). PFHpA was detected in two samples at 0.06 and 0.20 ng m^–3^. Overall, the total levels of extractable PFASs in the sampled flue gases were comparable during Sludge:MSWI and MSWI (3.4–5.6 and 4.1–5.6 ng m^–3^, respectively).

## Environmental Implications

**Figure 3:**
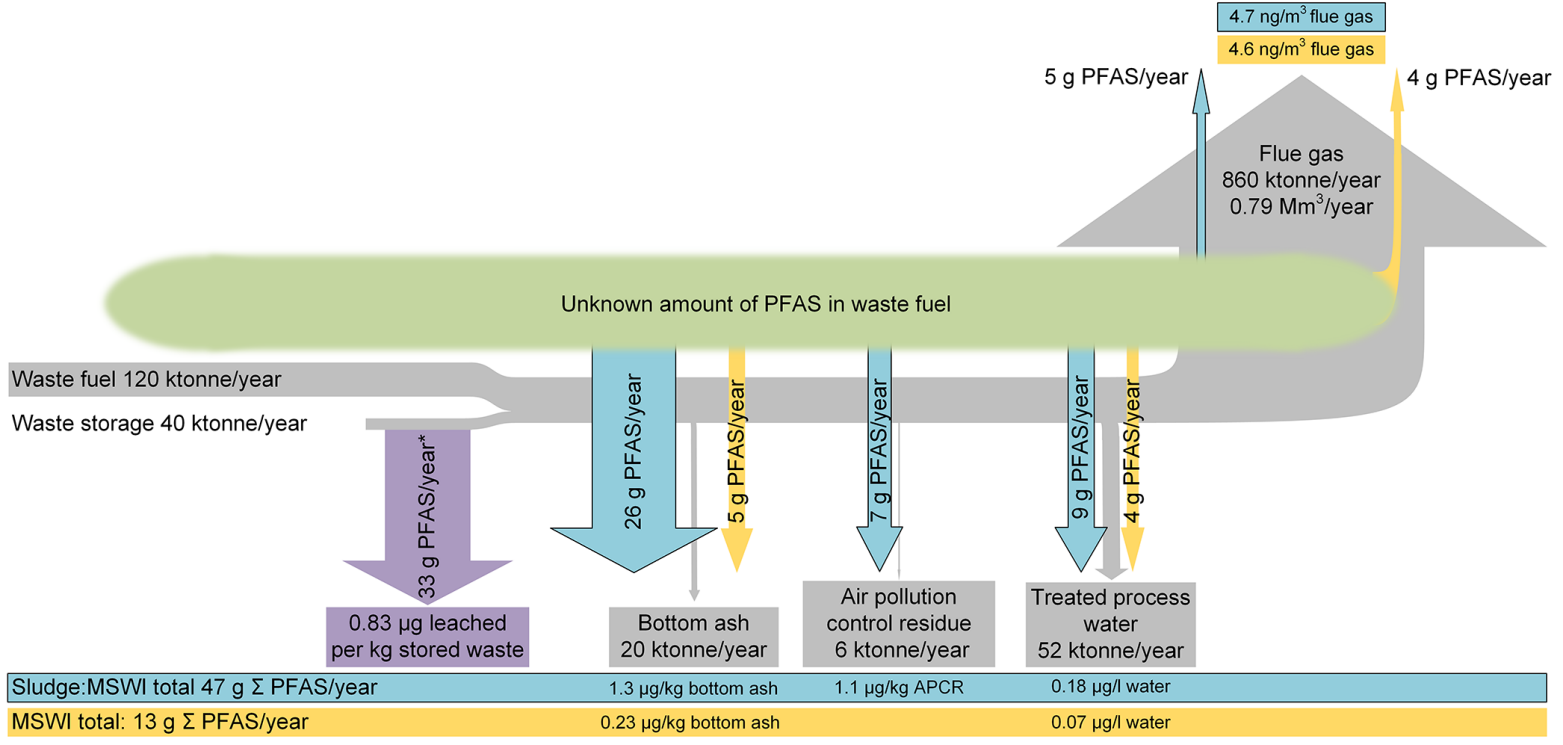
Mass flows on an annual basis, based on the findings in this study, of PFASs from the waste-to-energy plant presented by the respective residue stream. Gypsum is not shown due to negligible quantities. MSWI: municipal solid waste incineration. *Release of PFASs from waste storage is described in Björklund et al.^10^

The total annual release of PFASs during MSWI was estimated to be between 7 and 20 g, or 0.07–0.1 μg per kg of incinerated waste (Figure 3). In contrast, the annual release obtained when adding sludge to the waste fuel mix (i.e., the Sludge:MSWI case) was almost 4 times higher, at 11–56 g per year (0.07–0.4 μg per kg waste). This difference stems mainly from the higher abundance of PFASs in bottom ash during Sludge:MSWI compared to MSWI (Figure 3). In both cases, short-chain PFCAs were dominant, corresponding to 99% of the total extractable PFAS content during Sludge:MSWI, and 75% during MSWI. Annual release via leachate from the temporary waste stockpile at the same plant was investigated in a previous study and estimated to be 33 g per year.^10^ As a comparison, the amount of PFASs released via fly ash and bottom ash from three waste incineration plants in China was estimated to be 0.79–6.8 kg per year, or 0.49–4.6 μg kg^–1^ incinerated waste.^11^ However, when including leachates from waste stockpiles, the amount of PFASs released increased to 3.6–391 kg per year (21–65 μg kg^–1^ incinerated waste), compared to 0.05–0.08 kg per year (0.29–0.50 μg kg^–1^ incinerated waste) in the present study.

The importance of safe disposal of PFAS-containing waste has been highlighted by several researchers.^14−16,22^ Nevertheless, few studies have examined the fate and occurrence of PFASs in residues from MSW incineration. Until recently, the fate of PFASs in incineration was only considered in lab-scale studies investigating the degradation of PFASs for single compounds or materials under highly controlled conditions.^15^ For example, Watanabe et al. investigated the thermal degradation of PFASs during the regeneration of granular activated carbon and found that short-chain PFCAs were more thermally stable than long-chain PFCAs, and PFCAs were more stable than PFSAs.^23^ Conversely, Xiao et al. reported a higher thermal stability for PFSAs in comparison to PFCAs and increasing stability with increasing number of perfluorinated carbons.^24^ Moreover, lab-scale studies investigating the thermal degradation of fluoropolymers have obtained mixed results on the potential release of PFASs from MSW incineration, which could indicate that combustion conditions play an important role.^16^

These conflicting results highlight the urgent need for further research to bridge the gap between lab-scale and full-scale studies and better understand the fate of PFASs in waste incineration. This should include an analysis of how PFASs are distributed in internal residual streams of WtE plants as well as the removal efficiency of PFASs in flue gas treatment, topics which will be addressed in follow-up studies to this paper.

## Supporting Information Available

Additional experimental information, including details on plant operation, flue gas sampling method, quality assurance, and analytical parameters (PDF)
