# Supplementary material for: Emission of Per- and Polyfluoroalkyl Substances from a Waste-to-Energy Plant—Occurrence in Ashes, Treated Process Water, and First Observation in Flue Gas
Source: Environ Sci Technol. 2023 Jun 15;57(27):10089–95. doi: 10.1021/acs.est.2c08960 (PMC10339719; doi:10.1021/acs.est.2c08960)
Supplement: Supplementary file 1 — es2c08960_si_001.pdf [file es2c08960_si_001.pdf]

Supporting Information

Emission of per- and polyfluoroalkyl substances from a waste-to-energy plant – occurrence in ashes, treated process water, and first observation in flue gas

Sofie Björklund[a.b], Eva Weidemann[a], Stina Jansson[a] *

[a] Department of Chemistry, Umeå University, SE-901 87 Umeå, Sweden

[b] Industrial Doctoral School, Umeå University, SE-901 87 Umeå, Sweden

* Corresponding author: stina.jansson@umu.se

Number of pages: 17

Number of figures: 1

Number of tables: 12

Section 1: Detailed plant description (p. S2-S3)

Section 2: Flue gas sampling (p. S5)

Section S1. Detailed plant description.

The waste incineration plant is used for energy recovery from waste, with a 150-400 km uptake area. The waste fuel, except for a portion of waste that require pre-crushing, is delivered directly into the waste bunker to reduce need for handling from a hygienic and logistic perspective. The plant layout can be found in Figure 1 in the main article. A detailed description of the plant operation is given below:

To distribute moisture content and break up mono-fractions, manual fuel mixing is performed in the waste bunker using the waste handling crane. The crane is also used for adding approximately four 5-ton loads of fuel to the fuel hopper each hour, from where the moving grate boiler is fed continuously by a hydraulic pusher system.

After the incineration (minimum 2 s residence time at 850 °C in accordance to Swedish law, see SFS 2013:253 §32) the hot flue gases go through two empty passes to reduce temperature (from > 800 °C to ca 600 °C) before reaching the superheaters, where the heat is transferred to superheated steam which is used for electricity and district heat production. After the superheater, and an economizer, activated carbon is added to the warm flue gases (220 °C) which are then allowed to pass through textile filters to remove dioxins and ash. This ash is combined with the superheater ash, and constitute the dry part of the air pollution control residue (APCR). After the textile filters, the flue gas passes a second economizer before being quenched with water to reduce the temperature. After the quench, the flue gases pass through the acid scrubber, where water is used to wash out the HCl, NH_3 and Hg from the flue gases. A portion of water from the acid scrubber is directed to the process water treatment, while the majority is recirculated in the scrubber. The flue gases continue into the SO_2 scrubber, where the flue gases are sprayed with a slaked lime mixture ($Ca(OH)_2$) that forces the SO_2 from the gas phase to form gypsum ($CaSO_4$) which is collected as a separate waste stream. After the SO_2 scrubber, the flue gases are led through a condensate scrubber, removing some of the excess water and the remaining heat. Lastly, the flue gases are reheated slightly before they are released through the stack. All stack gas measuring equipment are placed directly before the gases are released into the air.

The process water treatment consists of two main sections – the first section removes heavy metals and other contaminants from the HCl-scrubber water while the second section removes harmful substances from the final flue gas condensate step. The first section, which has a flow that varies between 1.5 to 2.0 $m^3 \cdot h^{-1}$, consists of pH-adjustment, a CO_2-stripper, precipitation of heavy metals using TMT-15 (15 % water solution of $C_3N_3S_3Na_3$; CAS 17766-26-6, Algol Chemicals, Esbo, Finland), flocculation using a sulphur group functionalized organic polymer Kurifloc 6504 (Kurita Europe, Mannheim, Germany) and finally a lamella clarifier. Water from the first section is mixed with the water from the condensate scrubber and go through another set of precipitation, flocculation and lamella clarification at a rate between 4 to 14 $m^3 \cdot h^{-1}$. The setup is constructed to allow the considerably more contaminated HCl-scrubber condensate to be treated twice, even if a notable dilution occurs when the final condensate is introduced into the process. The final stage of the water treatment process is a sand filter. Solid phase waste from the process water treatment (from the precipitation steps and lamella clarifiers) is mixed with the ash from superheater and textile filters to form self-hardening APCR. The APCR is landfilled at a hazardous waste landfill.

During 2021, the plant logged no hours of operations with emissions exceeding legislative limits and had very good adherence to the environmental requirements outlined in Swedish law (SFS 2013:253). For example, CO did not exceed the 150 mg CO·Nm^{-3} limit for more than seven 10-minute periods/24 hours during the year (these are the terms of the legislation). The NOx emissions exceeded legislative limits (200 mg·Nm^{-3}) during five 30-minute periods, SO_2 emissions exceeded legislative limits (50 mg·Nm^{-3}) for thirty 30-minute periods, and TOC emissions exceeded legislative limits (10 mg·Nm^{-3}) for two 30-minute periods. The 2013:253 allows NOx, SO_2 and TOC emissions to exceed the boundary value for less than 3% of the running time, or 468 30-minute periods out of the 7800 hours of operating time. The plant operates within these limits, and in Table S1 the adherence to SFS 2013:253 with regard to emissions of metals, acids, ammonia and dioxins to recipient water and air through treated process water and flue gas is outlined. The emissions are below the legislative limits for all measured contaminants.

Table S1. Emissions of metals, acids, ammonia and dioxins in treated process water and flue gas from the waste incineration plant where sampling was conducted. Values were obtained from the facility's annual environmental report to the Swedish EPA.

	Treated process water (mg·L^{-1})		Flue gas (mg·Nm^{-3})*	
	Legislative limit	Average 2021	Legislative limit	Average 2021
As	0.15	0.007	0.5	0.0003
Cd	0.001	0.0001	0.03** (0.05)	0.00004
Co	-†	-†	0.5	0.00007
Cr	0.05	0.005	0.5	0.003
Cu	0.5	0.001	0.5	0.001
Hg	0.001	0.0002	0.03** (0.05)	0.002
Mn	-†	-†	0.5	0.002
Ni	0.2	0.004	0.5	0.004
Pb	0.05	0.0008	0.5	0.0006
Sb	-	-†	0.5	0.0002
Tl	0.05	0.0009	0.05	0.00006
V	-†	-†	0.5	0.0002
Zn	0.5	0.006	-†	-†
pH	6.5-9.0	8.1	-†	-†
HCL				0.18
HF				0.006
NH3				0.02
PCDD/F	0.3 (ng I-TEQ·L^{-1})	0.003 (ng I-TEQ·L^{-1})	0.1 (ng I-TEQ·Nm^{-3})	0.005 (ng I-TEQ·Nm^{-3})

* Nm^3: flue gas normalized to dry gas and 11 % O_2, validated according to SFS 2013:253

**: Locally enforced limit value, value from SFS 2013: 253 in brackets

†: Not measured in this compartment

Section S2. Flue gas sampling.

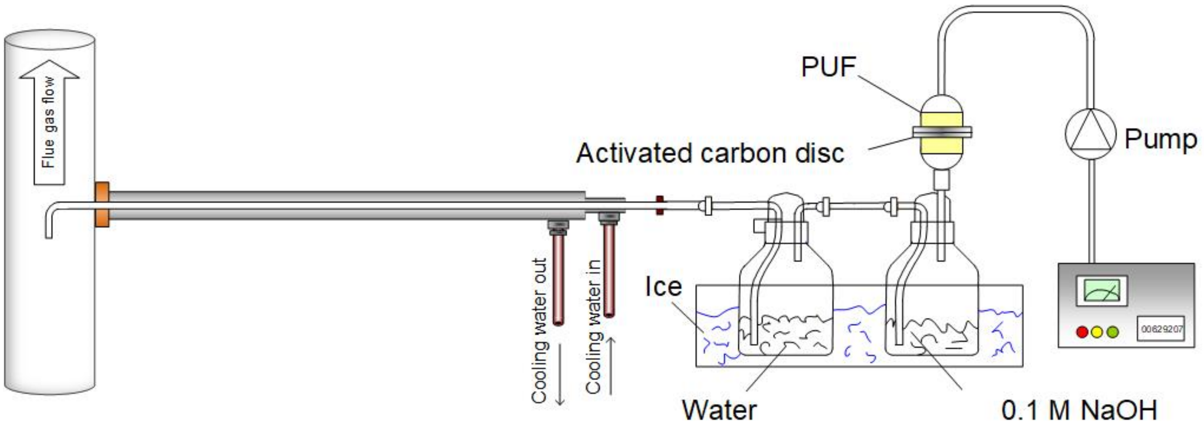

Figure S1. Flue gas sampling train.

Flue gas sampling was conducted in the stack during 6 hours per sampling occasion, at a rate of approximately 16 L min^{-1}. A total of 6 sampling occasions were performed, three during which the waste fuel mix incinerated was normal municipal solid waste, and three during which 5-8wt% of sludge from a wastewater treatment plant was added to the fuel mix.

The sampling train used was based on method EN 1948:1 (designed for dioxin sampling) (Fig. S1). The sampling train consists of a cooled glass probe (8 mm i.d.) which is inserted into the centre of the flue gas duct. The flue gases are led to a impinger bottle (2L) containing 250 mL of MilliQ water. The MilliQ water was spiked with 3 ng labelled standard prior to sampling to account for losses during sampling. The gases are then led to an impinger bottle (2L) containing 200 mL 0.1 M sodium hydroxide. The bottles are placed in an ice bath to promote condensation of water vapor present in the flue gas. Following the impinger bottles is a filter holder containing a pre-cleaned activated carbon disc (0.3 g; Futamura Chemical CO. LTD. Nagoya. Aichi. Japan) mounted between two polyurethane foam filters.

To facilitate comparison of levels in flue gas, the sampled flue gas volume was normalized to dry gas, 0°C and 1 atm pressure (see eq. S1).

Equation S1.

$$Normalized\ gas\ volume = V_{pump} \times p_{air} \times \frac{t_{0°C}}{t_{pump} \times t_{0°C}} \times (1 - \frac{\%H_2O}{100})$$

V_{pump} = Gas volume sampled (m^3)

p_{air} = Air pressure at sampling (atm)

t_{pump} = Temperature at sampling (°C)

$t_{0°C}$ = Temperature at 0°C (K)

$\%H_2O$ = % of H_2O in sampled flue gas

Table S2. Target compounds included in the analysis, their chemical formula, parent ion, quantification ion and the corresponding internal and recovery standard.

Acronym	Compound	Formula	Parent ion (m/z)	Quantification ion (m/z)	Qualification ion (m/z)	Internal standard
**PFCAs**	**Perfluorocarboxylic acids**					
PFBA	Perfluorobutanoic acid	$C_3F_7CO_2H$	212.98	168.99	-	M4PFBA
PFPeA	Perfluoropentanoic acid	$C_4F_9CO_2H$	262.98	218.99	-	M2PFHxA
PFHxA	Perfluorohexanoic acid	$C_5F_{11}CO_2H$	312.97	268.99	118.95	M2PFHxA
PFHpA	Perfluoroheptanoic acid	$C_6F_{13}CO_2H$	362.97	318.98	167.97	M2PFOA
PFOA	Perfluorooctanoic acid	$C_7F_{15}CO_2H$	412.97	368.98	168.97	M2PFOA
PFDA	Perfluorodecanoic acid	$C_9F_{19}CO_2H$	512.97	468.97	219.00	M2PFDA
PFDoDA	Perfluorododecanoic acid	$C_{11}F_{23}CO_2H$	612.97	568.96	168.96	M2PFDA
PFTeDA	Perfluorotetradecanoic acid	$C_{13}F_{27}CO_2H$	712.95	668.96	168.97	M2PFDA
**PFSAs**	**Perfluorosulphonic acids**					
PFBS	Perfluorobutanesulphonic acid	$C_4F_9SO_3H$	298.95	79.96	79.96	M3PFHxS
PFPeS	Perfluoropentanesulphonic acid	$C_5F_{11}SO_3H$	348.94	79.96	80.00	M3PFHxS
PFHxS	Perfluorohexanesulphonic acid	$C_6F_{13}SO_3H$	398.94	79.96	98.90	M3PFHxS
PFHpS	Perfluoroheptanesulphonic acid	$C_7F_{15}SO_3H$	448.94	98.96	79.96	M4PFOS
PFOS	Perfluorooctanesulphonic acid	$C_8F_{17}SO_3H$	498.93	98.96	79.96	M4PFOS
PFDoDS	Perfluorododecanesulphonic acid	$C_{12}F_{25}SO_3H$	698.92	98.96	79.96	M4PFOS
**FTSAs**	**Fluorotelomer sulphonic acids**					
6:2 FTSA	6:2 fluorotelomersulphonic acid	$C_8F_{13}H_4SO_3H$	426.97	80.96	81.00	M2-6:2FTSA
8:2 FTSA	8:2 fluorotelomersulphonic acid	$C_{10}F_{17}H_4SO_3H$	526.96	80.96	81.00	M2-6:2FTSA
**diPAPs**	**Polyfluoroalkyl phosphoric acid diesters**					
6:2diPAP	6:2 Fluorotelomer phosphate diester	$C_{16}H_9F_{26}O_4P$	788.98	442.97	96.97	M4PFOS
8:2diPAP	8:2 Fluorotelomer phosphate diester	$C_{20}H_9F_{34}O_4P$	988.97	542.97	96.97	M4PFOS

Acronym	Compound	Parent ion (m/z)	Quantification ion (m/z)	Recovery standard
**Internal standards**				
M4PFBA	Perfluoro-n-[1,2,3,4-$^{13}C_4$] butanoic acid	215.97	171.99	M8PFOA
M2PFHxA	Perfluoro-n-[1,2-$^{13}C_2$] hexanoic acid	314.97	269.99	M4PFOA
M2PFOA	Perfluoro-n-[1,2-$^{13}C_4$] octanoic acid	414.97	371.98	M8PFOA
M2PFDA	Perfluoro-n-[1,2-$^{13}C_2$] decanoic acid	514.97	469.97	M8PFOA
M3PFHxS	Perfluoro-1-[(1,2,3-$^{13}C_3$] hexanesulphonic acid	401.95	98.96	M8PFOS
M4PFOS	Perfluoro-1-[1,2,3,4-$^{13}C_4$]octanesulphonic acid	502.95	98.96	M8PFOS
M2-6:2FTSA	1H,1H,2H,2H-perfluoro-1-[1,2-$^{13}C_2$]-octane sulphonic acid	428.98	80.96	M8PFOS

**Recovery standards**			
M4PFOA	Perfluoro-n-[1,2,3,4-$^{13}C_4$] octanoic acid	416.98	171.99
M8PFOS	Perfluoro-1-[$^{13}C_8$]octanesulphonic acid	506.96	98.96

*Table S3.Concentrations of PFAS in all samples. Concentrations below the method detection limit is marked by <LOD. Numbers in italics are above LOD but below limit of quantification (LOQ). Results <LOD were treated as zero when calculating sums and averages. *Compound was quantified using the internal standard closest in retention time and levels should be considered semi-quantitative.*

	Unit	PFBA	PFPeA*	PFHxA	PFHpA*	PFOA	PFDA*	PFBS*	PFOS	Sum
Flue gas MSWI day 1		3.8	<LOD	0.22	<LOD	0.20	<LOD	<LOD	<LOD	4.2
Flue gas MSWI day 2		3.6	<LOD	0.25	<LOD	0.16	0.05	*0.03*	<LOD	4.1
Flue gas MSWI day 3	ng/m^3	2.8	<LOD	1.9	*0.23*	0.40	0.13	*0.03*	0.07	5.6
Average		3.4	<LOD	0.79	*0.08*	0.25	0.06	0.02	0.02	4.6
Flue gas Sludge:MSWI day 1		2.6	<LOD	0.39	<LOD	0.23	0.10	0.10	<LOD	3.4
Flue gas Sludge:MSWI day 2		2.5	<LOD	2.0	*0.21*	0.61	0.23	0.07	<LOD	5.6
Flue gas Sludge:MSWI day 3	ng/m^3	2.7	<LOD	0.89	*0.07*	0.79	0.20	0.24	<LOD	4.9
Average		2.6	<LOD	1.1	*0.09*	0.54	0.18	0.14	<LOD	4.7
Effluent water MSWI day 1		<LOD	<LOD	57	2.5	2.0	<LOD	<LOD	<LOD	62
Effluent water MSWI day 2		<LOD	<LOD	60	1.8	1.7	<LOD	<LOD	<LOD	63
Effluent water MSWI day 3	ng/L	<LOD	<LOD	94	<LOD	3.2	<LOD	<LOD	<LOD	97
Average		<LOD	<LOD	70	1.4	2.3	<LOD	<LOD	<LOD	74
Effluent water Sludge:MSWI day 1		8.8	3.5	141	4.0	4.3	0.94	<LOD	<LOD	163
Effluent water Sludge:MSWI day 2		13	3.0	132	4.3	5.9	1.6	<LOD	<LOD	160
Effluent water Sludge:MSWI day 3	ng/L	10	4.3	190	7.6	6.6	1.3	<LOD	<LOD	220
Average		11	3.6	154	5.3	5.6	1.3	<LOD	<LOD	181
Bottom ash MSWI day 1		<LOD	<LOD	0.16	<LOD	<LOD	<LOD	<LOD	<LOD	0.16
Bottom ash MSWI day 2		<LOD	<LOD	<LOD	<LOD	<LOD	<LOD	<LOD	<LOD	<LOD
Bottom ash MSWI day 3	ng/g	<LOD	<LOD	<LOD	<LOD	*0.54*	<LOD	<LOD	<LOD	0.54
Average		<LOD	<LOD	0.05	<LOD	0.18	<LOD	<LOD	<LOD	0.23
Bottom ash Sludge:MSWI day 1		1.4	<LOD	<LOD	<LOD	<LOD	<LOD	<LOD	<LOD	1.4
Bottom ash Sludge:MSWI day 2		0.81	<LOD	<LOD	<LOD	<LOD	<LOD	<LOD	<LOD	0.81
Bottom ash Sludge:MSWI day 3	ng/g	1.5	<LOD	<LOD	<LOD	<LOD	<LOD	<LOD	<LOD	1.5
Average		1.3	<LOD	<LOD	<LOD	<LOD	<LOD	<LOD	<LOD	1.3
APCR MSWI day 1		<LOD	<LOD	<LOD	<LOD	<LOD	<LOD	<LOD	<LOD	<LOD
APCR MSWI day 2		<LOD	<LOD	<LOD	<LOD	<LOD	<LOD	<LOD	<LOD	<LOD
APCR MSWI day 3	ng/g	<LOD	<LOD	<LOD	<LOD	<LOD	<LOD	<LOD	<LOD	<LOD
Average		<LOD	<LOD	<LOD	<LOD	<LOD	<LOD	<LOD	<LOD	<LOD
APCR Sludge:MSWI day 1	ng/g	1.2	<LOD	0.11	<LOD	<LOD	<LOD	<LOD	<LOD	1.3

APCR Sludge:MSWI day 2		0.99	<LOD	<LOD	<LOD	<LOD	<LOD	<LOD	<LOD	1.0
APCR Sludge:MSWI day 3		1.1	<LOD	<LOD	<LOD	<LOD	<LOD	<LOD	<LOD	1.1
Average		1.1	<LOD	0.04	<LOD	<LOD	<LOD	<LOD	<LOD	1.1
Gypsum MSWI day 1	ng/g	<LOD	<LOD	<LOD	<LOD	<LOD	<LOD	<LOD	<LOD	<LOD
Gypsum MSWI day 2		<LOD	<LOD	<LOD	<LOD	<LOD	<LOD	<LOD	<LOD	<LOD
Gypsum MSWI day 3		<LOD	<LOD	<LOD	<LOD	<LOD	<LOD	<LOD	<LOD	<LOD
Average		<LOD	<LOD	<LOD	<LOD	<LOD	<LOD	<LOD	<LOD	<LOD
Gypsum Sludge:MSWI day 1	ng/g	<LOD	<LOD	0.31	<LOD	<LOD	<LOD	<LOD	<LOD	0.31
Gypsum Sludge:MSWI day 2		<LOD	<LOD	0.17	<LOD	<LOD	<LOD	<LOD	<LOD	0.17
Gypsum Sludge:MSWI day 3		<LOD	<LOD	0.26	<LOD	<LOD	<LOD	<LOD	<LOD	0.26
Average		<LOD	<LOD	0.25	<LOD	<LOD	<LOD	<LOD	<LOD	0.25

Table S4. Limit of detection per sample matrix.

	Effluent water	APCR	Bottom ash	Gypsum	Flue gas
	ng/l	ng/g	ng/g	ng/g	ng/m^3
**PFBA**	1.2	0.25	0.24	0.23	0.06
**PFPeA**	0.12	0.06	0.06	0.06	0.01
**PFHxA**	0.12	0.06	0.06	0.06	0.01
**PFHpA**	0.20	0.05	0.05	0.05	0.01
**PFOA**	0.82	0.17	0.17	0.16	0.04
**PFDA**	0.31	0.06	0.06	0.06	0.01
**PFDoDA**	3.1	0.64	0.62	0.59	0.15
**PFTeDA**	1.7	0.36	0.35	0.34	0.10
**PFBS**	0.32	0.07	0.06	0.06	0.02
**PFPeS**	0.06	0.06	0.06	0.06	0.02
**PFHxS**	0.06	0.06	0.06	0.06	0.03
**PFHpS**	0.06	0.06	0.06	0.06	0.03
**PFOS**	0.09	0.06	0.06	0.06	0.02
**PFDoDS**	3.2	0.67	0.65	0.61	0.16
**6:2FTS**	1.7	0.35	0.34	0.32	0.08
**8:2FTS**	1.0	0.22	0.21	0.20	0.05
**6:2diPAP**	5.4	1.1	1.1	1.0	0.26
**8:2diPAP**	6.9	1.4	1.4	1.3	0.34

Table S5. Relative standard deviation of replicates per sample.

	PFBA	PFPeA	PFHxA	PFHpA	PFOA	PFDA
Bottom ash Sludge:MSWI day 1 (n=3)	17%	<LOD	<LOD	<LOD	<LOD	<LOD
Bottom ash MSWI day 1 (n=3)	<LOD	<LOD	21%	<LOD	<LOD	<LOD
APCR Sludge:MSWI day 1 (n=3)	10%	<LOD	49%	<LOD	<LOD	<LOD
APCR MSWI day 3 (n=3)	<LOD	<LOD	<LOD	<LOD	<LOD	<LOD
Gypsum Sludge:MSWI day 1 (n=3)	<LOD	<LOD	13%	<LOD	<LOD	<LOD
Gypsum MSWI day 1 (n=3)	<LOD	<LOD	<LOD	<LOD	<LOD	<LOD
Effluent water Sludge:MSWI day 1 (n=3)	6%	6%	6%	8%	2%	10%
Effluent water MSWI day 2 (n=2)	<LOD	<LOD	7%	11%	8%	<LOD

Table S6. Concentration of PFAS in field blanks.

	Unit	PFBA	PFPeA	PFHxA	PFHpA	PFOA	PFDA	PFDoDA	PFTeDA	PFBS
**Effluent water MSWI**	ng/L	<LOD	<LOD	<LOD	<LOD	0.83	<LOD	<LOD	<LOD	<LOD
**Effluent water Sludge:MSWI**		4.8	<LOD	0.31	<LOD	4.5	<LOD	<LOD	<LOD	<LOD
**Flue gas MSWI**	ng/m^3	0.90	<LOD	0.02	<LOD	*0.06*	<LOD	<LOD	<LOD	0.03
**Flue gas Sludge:MSWI**		0.64	<LOD	0.06	<LOD	0.29	0.16	<LOD	<LOD	0.16
	**Unit**	**PFPeS**	**PFHxS**	**PFHpS**	**PFOS**	**PFDoDS**	**6:2FTS**	**8:2FTS**	**6:2diPAP**	**8:2diPAP**
**Effluent water MSWI**	ng/L	<LOD	<LOD	<LOD	<LOD	<LOD	<LOD	<LOD	<LOD	<LOD
**Effluent water Sludge:MSWI**		<LOD	<LOD	<LOD	<LOD	<LOD	<LOD	<LOD	<LOD	<LOD
**Flue gas MSWI**	ng/m^3	<LOD	<LOD	<LOD	<LOD	<LOD	<LOD	<LOD	<LOD	<LOD
**Flue gas Sludge:MSWI**		<LOD	<LOD	<LOD	<LOD	<LOD	<LOD	<LOD	<LOD	<LOD

Tabell S7. Concentration of PFAS in procedural blanks (ng·L⁻¹).

Compound	PFBA	PFPeA	PFHxA	PFHpA	PFOA	PFDA	PFDoDA	PFTeDA	PFBS	PFPeS
**Blank 1**	1.6	<LOD	<LOD	<LOD	<LOD	<LOD	<LOD	<LOD	<LOD	<LOD
**Blank 2**	7.4	<LOD	<LOD	<LOD	<LOD	<LOD	<LOD	<LOD	<LOD	<LOD
**Blank 3**	<LOD	<LOD	<LOD	<LOD	<LOD	<LOD	<LOD	<LOD	<LOD	<LOD
**Blank 4**	<LOD	<LOD	<LOD	<LOD	<LOD	<LOD	<LOD	<LOD	<LOD	<LOD
**Blank 5**	<LOD	<LOD	<LOD	<LOD	<LOD	<LOD	<LOD	<LOD	<LOD	<LOD
**Blank 6**	<LOD	<LOD	<LOD	<LOD	<LOD	<LOD	<LOD	<LOD	<LOD	<LOD
**Blank 7**	<LOD	<LOD	<LOD	<LOD	<LOD	<LOD	<LOD	<LOD	<LOD	<LOD
**Blank 8**	<LOD	<LOD	<LOD	<LOD	<LOD	<LOD	<LOD	<LOD	<LOD	<LOD
**Blank 9**	<LOD	<LOD	<LOD	<LOD	<LOD	<LOD	<LOD	<LOD	<LOD	<LOD
**Blank 10**	<LOD	<LOD	<LOD	<LOD	<LOD	<LOD	<LOD	<LOD	<LOD	<LOD
**Blank 11**	<LOD	<LOD	<LOD	<LOD	<LOD	<LOD	<LOD	<LOD	<LOD	<LOD
**Blank 12**	<LOD	<LOD	<LOD	<LOD	<LOD	<LOD	<LOD	<LOD	<LOD	<LOD
**Blank 13**	<LOD	<LOD	<LOD	<LOD	<LOD	<LOD	<LOD	<LOD	<LOD	<LOD
**Blank 14**	<LOD	<LOD	<LOD	<LOD	<LOD	<LOD	<LOD	<LOD	<LOD	<LOD
**Blank 15**	<LOD	<LOD	<LOD	<LOD	<LOD	<LOD	<LOD	<LOD	<LOD	<LOD
**Blank 16**	<LOD	<LOD	<LOD	<LOD	<LOD	<LOD	<LOD	<LOD	<LOD	<LOD
**Blank 17**	<LOD	<LOD	<LOD	<LOD	<LOD	<LOD	<LOD	<LOD	<LOD	<LOD
**Blank 18**	<LOD	<LOD	<LOD	<LOD	<LOD	<LOD	<LOD	<LOD	<LOD	<LOD

**Compound**	**8:2diPAP**	**6:2diPAP**	**8:2FTS**	**6:2FTS**	**PFDoDS**	**PFOS**	**PFHpS**	**PFHxS**
**Blank 1**	<LOD	<LOD	<LOD	<LOD	<LOD	<LOD	<LOD	<LOD
**Blank 2**	<LOD	<LOD	<LOD	<LOD	<LOD	<LOD	<LOD	<LOD
**Blank 3**	<LOD	<LOD	<LOD	<LOD	<LOD	<LOD	<LOD	<LOD
**Blank 4**	<LOD	<LOD	<LOD	<LOD	<LOD	<LOD	<LOD	<LOD
**Blank 5**	<LOD	<LOD	<LOD	<LOD	<LOD	<LOD	<LOD	<LOD
**Blank 6**	<LOD	<LOD	<LOD	<LOD	<LOD	<LOD	<LOD	<LOD
**Blank 7**	<LOD	<LOD	<LOD	<LOD	<LOD	<LOD	<LOD	<LOD
**Blank 8**	<LOD	<LOD	<LOD	<LOD	<LOD	<LOD	<LOD	<LOD
**Blank 9**	<LOD	<LOD	<LOD	<LOD	<LOD	<LOD	<LOD	<LOD
**Blank 10**	<LOD	<LOD	<LOD	<LOD	<LOD	<LOD	<LOD	<LOD
**Blank 11**	<LOD	<LOD	<LOD	<LOD	<LOD	<LOD	<LOD	<LOD
**Blank 12**	<LOD	<LOD	<LOD	<LOD	<LOD	<LOD	<LOD	<LOD
**Blank 13**	<LOD	<LOD	<LOD	<LOD	<LOD	<LOD	<LOD	<LOD
**Blank 14**	<LOD	<LOD	<LOD	<LOD	<LOD	<LOD	<LOD	<LOD
**Blank 15**	<LOD	<LOD	<LOD	<LOD	<LOD	<LOD	<LOD	<LOD
**Blank 16**	<LOD	<LOD	<LOD	<LOD	<LOD	<LOD	<LOD	<LOD
**Blank 17**	<LOD	<LOD	<LOD	<LOD	<LOD	<LOD	<LOD	<LOD
**Blank 18**	<LOD	<LOD	<LOD	<LOD	<LOD	<LOD	<LOD	<LOD

Table S8. Average internal standard recoveries per sample matrix.

	PFBA	PFHxA	PFOA	PFDA	PFHxS	PFOS	6:2FTS
Blank	92% ±18%	83% ±11%	85% ±7%	89% ±18%	116% ±27%	108% ±23%	149% ±39%
APCR	85% ±17%	78% ±12%	82% ±4%	79% ±20%	115% ±21%	110% ±23%	117% ±26%
Bottom ash	76% ±5%	68% ±7%	80% ±5%	70% ±9%	116% ±19%	104% ±18%	103% ±25%
Effluent water	64% ±25%	72% ±29%	71% ±20%	66% ±22%	104% ±30%	92% ±34%	100% ±49%
Gypsum	104% ±48%	102% ±49%	80% ±15%	96% ±34%	128% ±42%	113% ±34%	131% ±52%
MilliQ Flue gas	98% ±14%	91% ±12%	84% ±13%	92% ±16%	90% ±13%	86% ±12%	180% ±60%
NaOH Flue gas	82% ±15%	74% ±13%	78% ±18%	62% ±24%	76% ±14%	61% ±21%	121% ±28%
Filter Flue gas	47% ±21%	42% ±23%	45% ±20%	44% ±23%	50% ±34%	48% ±37%	133% ±67%
**Average**	81% ±17%	76% ±16%	76% ±12%	75% ±17%	99% ±24%	90% ±23%	129% ±24%

Table S9. Concentration in individual compartments of flue gas sampling train (ng·m^{-3})

PFHxS	PFPeS	PFBS	PFTeDA	PFDoDA	PFDA	PFOA	PFHpA	PFHxA	PFPeA	PFBA
<LOD	<LOD	0.04	<LOD	<LOD	*0.02*	0.08	<LOD	0.08	<LOD	0.67
<LOD	<LOD	<LOD	<LOD	<LOD	*0.03*	*0.06*	<LOD	0.20	<LOD	<LOD
<LOD	<LOD	0.06	<LOD	<LOD	0.05	*0.09*	<LOD	0.11	<LOD	1.9
<LOD	<LOD	*0.04*	<LOD	<LOD	0.05	*0.06*	<LOD	0.16	<LOD	0.81
<LOD	<LOD	<LOD	<LOD	<LOD	0.14	0.42	0.21	1.7	<LOD	0.73
<LOD	<LOD	*0.04*	<LOD	<LOD	*0.03*	*0.13*	<LOD	0.16	<LOD	0.94
<LOD	<LOD	<LOD	<LOD	<LOD	0.07	0.29	<LOD	0.27	<LOD	<LOD
<LOD	<LOD	*0.03*	<LOD	<LOD	0.07	*0.12*	0.07	0.43	<LOD	1.1
<LOD	<LOD	*0.05*	<LOD	<LOD	0.06	0.15	<LOD	0.19	<LOD	0.87
<LOD	<LOD	<LOD	<LOD	<LOD	<LOD	<LOD	<LOD	0.05	<LOD	2.3
<LOD	<LOD	<LOD	<LOD	<LOD	<LOD	*0.06*	<LOD	0.10	<LOD	1.4
<LOD	<LOD	<LOD	<LOD	<LOD	<LOD	*0.14*	<LOD	0.06	<LOD	<LOD
<LOD	<LOD	<LOD	<LOD	<LOD	<LOD	*0.06*	<LOD	0.04	<LOD	2.1
<LOD	<LOD	<LOD	<LOD	<LOD	*0.05*	*0.10*	<LOD	0.22	<LOD	1.5
<LOD	<LOD	*0.03*	<LOD	<LOD	<LOD	<LOD	<LOD	<LOD	<LOD	<LOD
<LOD	<LOD	<LOD	<LOD	<LOD	<LOD	*0.08*	<LOD	0.12	<LOD	2.1
<LOD	<LOD	<LOD	<LOD	<LOD	0.13	0.32	0.23	1.8	<LOD	0.79
<LOD	<LOD	*0.03*	<LOD	<LOD	<LOD	<LOD	<LOD	<LOD	<LOD	<LOD
<LOD	<LOD	0.02	<LOD	<LOD	0.10	*0.08*	<LOD	0.02	<LOD	<LOD
<LOD	<LOD	<LOD	<LOD	<LOD	*0.03*	<LOD	<LOD	<LOD	<LOD	<LOD
<LOD	<LOD	0.14	<LOD	<LOD	*0.03*	0.21	<LOD	0.04	<LOD	0.64
<LOD	<LOD	<LOD	<LOD	<LOD	<LOD	*0.06*	<LOD	0.02	<LOD	0.90
<LOD	<LOD	<LOD	<LOD	<LOD	<LOD	<LOD	<LOD	<LOD	<LOD	<LOD
<LOD	<LOD	0.03	<LOD	<LOD	<LOD	<LOD	<LOD	<LOD	<LOD	<LOD

Sample	Compartment	PFOS	PFHpS
**Sludge:MSWI day 1**	**NaOH**	<LOD	<LOD
**Sludge:MSWI day 1**	**MQ**	<LOD	<LOD
**Sludge:MSWI day 1**	**filter**	<LOD	<LOD
**Sludge:MSWI day 2**	**NaOH**	<LOD	<LOD
**Sludge:MSWI day 2**	**MQ**	<LOD	<LOD
**Sludge:MSWI day 2**	**filter**	<LOD	<LOD
**Sludge:MSWI day 3**	**NaOH**	<LOD	<LOD
**Sludge:MSWI day 3**	**MQ**	<LOD	<LOD
**Sludge:MSWI day 3**	**filter**	<LOD	<LOD
**MSWI day 1**	**NaOH**	<LOD	<LOD
**MSWI day 1**	**MQ**	<LOD	<LOD
**MSWI day 1**	**filter**	<LOD	<LOD
**MSWI day 2**	**NaOH**	<LOD	<LOD
**MSWI day 2**	**MQ**	<LOD	<LOD
**MSWI day 2**	**filter**	<LOD	<LOD
**MSWI day 3**	**NaOH**	*0.01*	<LOD
**MSWI day 3**	**MQ**	0.06	<LOD
**MSWI day 3**	**filter**	<LOD	<LOD
**FB Sludge:MSWI**	**NaOH**	<LOD	<LOD
**FB Sludge:MSWI**	**MQ**	<LOD	<LOD
**FB Sludge:MSWI**	**filter**	<LOD	<LOD
**FB MSWI**	**NaOH**	<LOD	<LOD
**FB MSWI**	**MQ**	<LOD	<LOD
**FB MSWI**	**filter**	<LOD	<LOD

Table S10. Flue gas sampling parameters.

Parameter	MSWI (min-max)	Sludge:MSWI (min-max)
Air pressure (atm)	0.99–1.0	0.98–0.99
Pump temperature (°C)	20	20
%H_2O wet gas	13–20	5.7–10
%CO_2 wet gas	7.8–16	8.7-9.32
%O_2 wet gas	6.9–8.0	8.1–8.4

Table S11. LC-MS instrument parameters.

Parameter	Value
**Gas Temp (°C)**	250
**Gas Flow (l/min)**	5
**Nebulizer (psig)**	30
**SheathGasTemp (°C)**	350
**SheathGasFlow (l/min)**	12
**Injection volume (µL)**	10.0

Table S12. LC mobile phase program.

	Time	A (2 mM NH_4Ac in MeOH)	B (2 mM NH_4Ac inH_2O)	Flow
**1**	2.00 min	70.00 %	30.00 %	0.5 mL/min
**2**	14.00 min	0.00 %	100.00 %	0.5 mL/min
**3**	17.00 min	0.00 %	100.00 %	0.5 mL/min
